# Endothelin-Converting Enzyme-1 (ECE-1) Is Post-Transcriptionally Regulated by Alternative Polyadenylation

**DOI:** 10.1371/journal.pone.0083260

**Published:** 2014-01-31

**Authors:** Alison R. Whyteside, Anthony J. Turner, Daniel W. Lambert

**Affiliations:** 1 Institute of Molecular and Cellular Biology, University of Leeds, Leeds, United Kindom; 2 Integrated Biosciences, School of Clinical Dentistry, University of Sheffield, Sheffield, United Kingdom; 3 CR-UK/YCR Sheffield Cancer Centre, University of Sheffield, Sheffield, United Kingdom; Southern Illinois University School of Medicine, United States of America

## Abstract

Endothelin-converting enzyme-1 (ECE-1) is the enzyme predominantly responsible for producing active endothelin-1 (ET-1), a mitogenic peptide implicated in the aetiology of a number of diseases, including cancer. Elevated levels of ECE-1 have been observed in a range of malignancies, with high expression conferring poor prognosis and aiding the acquisition of androgen independence in prostate cancer. The mechanisms regulating the expression of ECE-1 in cancer cells are poorly understood, hampering the development of novel therapies targeting the endothelin axis. Here we provide evidence that the expression of ECE-1 is markedly inhibited by its 3′UTR, and that alternative polyadenylation (APA) results in the production of ECE-1 transcripts with truncated 3′UTRs which promote elevated protein expression. Abolition of the ECE-1 APA sites reduced protein expression from a reporter vector in prostate cancer cells, suggesting these sites are functional. This is the first study to identify ECE-1 as a target for APA, a regulatory mechanism aberrantly activated in cancer cells, and provides novel information about the mechanisms leading to ECE-1 overexpression in malignant cells.

## Introduction

ET-1 is a potent mitogen for a variety of cell types, including vascular smooth muscle cells, fibroblasts and endothelial cells, and is able to coordinate the proliferative effects of other peptide growth factors [1]. ET-1 binds to one of two G-protein coupled receptors, ET_A_R and ET_B_R, leading to activation of downstream signalling cascades promoting proliferation and chemotaxis [2]. Furthermore, ET-1 appears to stimulate pro-angiogenic factors, such as vascular endothelial growth factor (VEGF) [3]. The endothelin system has been implicated in the pathobiology of numerous human cancers including those of the prostate, lung, breast, colon and cervix, and plays a role in the aetiology of other pathologies such as hepatic fibrosis and atherosclerosis [4].

ET-1 is generated via processing of inactive big-ET-1 by endothelin-converting enzyme-1 (ECE-1). ECE-1 exists as four isoforms, ECE-1a to ECE-1d, which are generated from a single gene by the use of alternative promoters. The isoforms differ only in their N-terminal regions; the majority of exons and the 3′UTR are common to all four isoforms [5]. The isoforms exhibit similar catalytic activities but differ in their subcellular localisations [6]. ECE-1 is abundantly and widely expressed [7], implying an important physiological role. ECE-1 is upregulated in a number of cancers, including prostate cancer, leading to increased levels of ET-1 peptide [8], [9]. The mechanisms underlying the observed upregulation, however, remain to be elucidated.

Previous studies have provided evidence that ECE-1 may be subject to post-transcriptional regulation in hepatic stellate cells and vascular endothelial cells [10], [11]. Post-transcriptional regulation, including the processing, export, localisation, turnover and translation of messenger RNAs (mRNAs), is increasingly recognised as a major means of altering gene expression levels [12]. Many of these mechanisms of post-transcriptional regulation are directed by sequences or motifs present in the 3′ untranslated region (3′UTR) of transcripts, which may be targeted by regulatory molecules including proteins and microRNA. MicroRNAs (miRNAs) are small, non-coding RNA molecules which regulate expression of target genes by binding to complementary sequences in the 3′ UTR and causing transcript degradation or inhibiting translation [13], [14]. Widespread dysregulation of miRNA expression has been identified in numerous diseases, including prostate cancer [15]. Furthermore, widespread truncation of 3′UTR has recently been reported as a feature of cancer cells, suggesting aberrant post-transcriptional regulation of gene expression may be a critical contributing factor to malignant transformation and/or disease progression.

In this study we present analysis of the post-transcriptional regulation of ECE-1 and its implications in prostate cancer. We provide evidence that the ECE-1 3′UTR is able to regulate the expression of ECE-1 and identify the presence of truncated transcripts generated from alternative polyadenylation in cancer cells.

## Experimental Procedures

### Materials

RWPE-1 prostate epithelial cells were purchased from ATCC via LGC Standards. PC-3 cells were kindly provided by Prof. Norman Maitland (University of York, UK; [16]). CHO cells were originally obtained from ECACC.

The pMIR-REPORT Luciferase vector and pre-miR molecules were purchased from Ambion (Warrington, UK). The pRL-TK vector, pSV-β-galactosidase vector and dual-luciferase reporter assay system were from Promega (Southampton, UK). The pcDNA3 mammalian expression vector, Lipofectamine2000, Oligofectamine and keratinocyte serum-free medium (SFM) were from Invitrogen (Paisley, UK).

The following antibodies were used at the following dilutions: monoclonal anti-V5 antibody 1∶500 (Invitrogen), goat polyclonal anti-ECE-1 1:1000 (R&D Systems), rabbit polyclonal anti-Upf1 1:200 (Santa Cruz) and monoclonal anti-β-actin 1∶10,000 (Sigma).

### Cell Culture

PC-3 cells were routinely cultured in Hams F-12 containing 7% (v/v) FBS and 2 mM L-glutamine. RWPE-1 cells were cultured in Keratinocyte-SFM containing L-glutamine supplemented with bovine pituitary extract (0.05 mg/ml) and epidermal growth factor (5 ng/ml) (Invitrogen). CHO cells were cultured in Hams F-12 containing 10% (v/v) FBS and 2 mM L-glutamine. Huh7 cells were cultured in DMEM containing 10% (v/v) FBS, non essential amino acids and 2 mM L-glutamine.

### Cloning of 3′UTR and Site-directed Mutagenesis

The full-length ECE-1 3′UTR (2710 nucleotides corresponding to bases 2534–5243 of NCBI accession NM_001113347) was amplified from human genomic DNA (BD Biosciences) using Phusion polymerase (NEB) and ligated into pMIR-REPORT Luciferase vector between the SpeI and HindIII sites. The insert was fully sequenced.

6 alternative polyadenylation (APA) sites were identified in the ECE-1 3′UTR. These were sequentially mutated by PCR. To remove the SV40polyA sequence from the miReport vector, a MluI site was introduced at either side of the SV40 sequence, the construct digested and subsequently re-ligated.

### 3′ RACE

Total RNA was isolated from PC-3 cells using the RNeasy kit (QIAgen). 1 µg total RNA was used to generate cDNA using Superscript II reverse transcriptase (Invitrogen) according to manufacturer’s instructions using TAP as a primer (GACTCGAGCAAGCTTCGATTTTTTTTTTTTTTTTT). 2 µl of the cDNA reaction was used as a template for first round PCR using an ECE-1-specific forward primer (GCTTTGCACAGGTCTGGTGCTC) and AP as reverse primer (GACTCGAGCAAGCTTCG). Nested PCR was then performed using a nested ECE-1-specific forward primer (AATTCCAAGGAGTTCTCAG) and MAP as reverse primer (AAGCTTCGATTTTTTTTTT). The PCR products were then A-tailed and ligated into pCR4-TOPO (Invitrogen) according to manufacturer’s instructions. Constructs were sequenced using M13 forward and reverse primers.

The ECE-1 UTR-specific sequence was then cloned into pMIR-REPORT Luciferase between the SpeI and MluI sites. To control for length, the reverse complement (RC) of the remaining UTR sequence was then cloned in between the MluI and HindIII sites.

### Construction of Expression Vectors

ECE-1c cDNA with an N-terminal V5 tag was ligated into the pcDNA3 expression vector between KpnI and XbaI sites. To make cDNA plus UTR, the full-length or truncated UTR was ligated in immediately downstream of the cDNA.

Huh7 and CHO cells were transfected with 5 µg DNA using Lipofectamine transfection reagent. PC-3 cells were transfected with 15 µg DNA using TransIT-Prostate Transfection Kit (Mirus). RWPE-1 cells were transfected with 5.4 µg DNA using TransIT-LT1 transfection reagent (Mirus).

### Luciferase Reporter Assays

Cells were co-transfected using Lipofectamine2000 with 500 ng empty luciferase reporter vector (miReport) or 500 ng 3′UTR-luciferase reporter vector and 100 ng pRL-TK Renilla luciferase control vector. 48 h after transfection, cells were rinsed in PBS, lysed in passive lysis buffer and analysed using the dual-luciferase reporter assay system according to manufacturer’s instructions.

Huh7 cells were co-transfected using Lipofectamine2000 with 500 ng 3′UTR-luciferase reporter vector, 300 ng pSV-β-galactosidase control vector and pre-miRNA or negative control miRNA #1 (final concentration 47.62 nM). 48 h after transfection, cells were rinsed in PBS, lysed in reporter lysis buffer and analysed using the dual-luciferase reporter assay system and the β-galactosidase enzyme assay system according to manufacturer’s instructions.

### Protein Extraction and Immunoblotting

Cells were lysed in triple detergent buffer containing protease inhibitors and benzonase nuclease. Proteins were separated on 3–8% tris-acetate gradient gels (Invitrogen), transferred to nitrocellulose membrane, blocked and incubated with primary antibodies overnight. After washing, membranes were incubated with HRP-conjugated secondary antibodies for 1 hour. Densitometrical analysis was performed using Aida v2.1 software.

### Quantitative Real-time PCR

Total RNA was isolated from PC-3 cells 48 h after transfection with V5 cDNA only or V5 cDNA plus full-length UTR using the RNeasy mini kit (Qiagen) and treated with DNase I (Invitrogen). cDNA was produced from 500 ng total RNA using SuperScript II reverse transcriptase (Invitrogen) with oligo(dT) primers (Promega) according to manufacturer’s instructions. 10 ng cDNA was then used as template per reaction. Quantitative real-time PCR was performed using 2× SensiMix SYBR & Fluoroscein kit (Bioline) and 0.5 µM each primer.

V5 forward CTA ACC CTC TCC TCG GTC TCG AT (in the V5 tag).

V5-ECE-1 reverse CCG GGG GCT GTG GAA GTT CAC (ECE-1 specific).

The amplified product of this primer pair results only from the reverse-transcribed V5-tagged ECE-1 construct. For each RNA sample, triplicates were analysed with each primer set. Relative ECE-1 RNA expression was normalised to neomycin for transfection efficiency, and endogenous U6.

Neomycin forward CTT GCT CCT GCC GAG AAA GT.

Neomycin reverse TTC GCT TGG TGG TCG AAT G.

U6 forward CTC GCT TCG GCA GCA CA.

U6 reverse AAC GCT TCA CGA ATT TGC GT.

## Results

### The Full-length 3′UTR of ECE-1 Significantly Reduces Luciferase Activity

**Figure 1:**
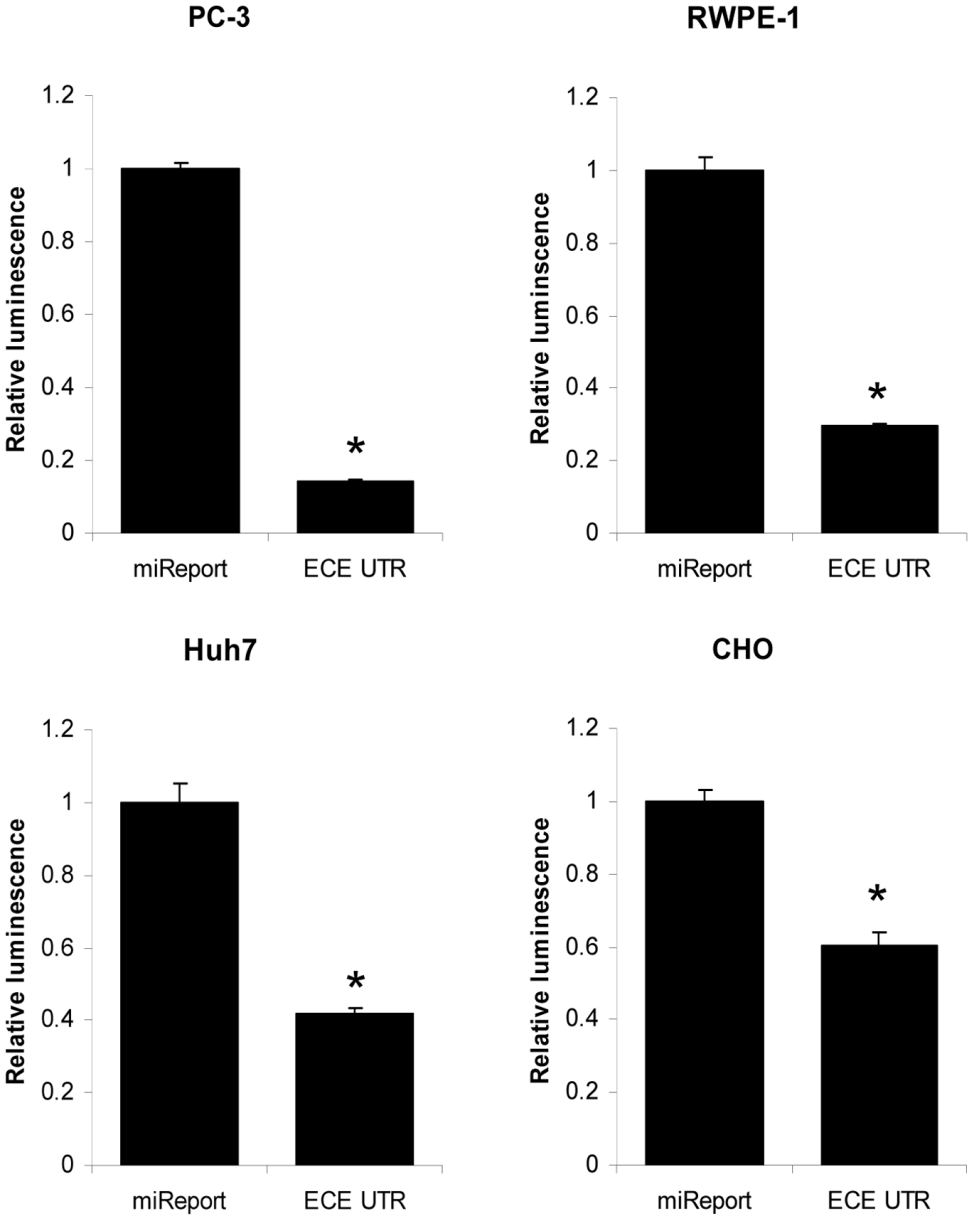
The full-length ECE-1 3′UTR significantly reduces luciferase activity. The full-length ECE-1 3′UTR was cloned into pMIR-REPORT luciferase vector (ECE UTR) and transfected into the cell lines indicated. Activities of Firefly and Renilla luciferase (endogenous control) were assayed 48 h post transfection. Data represent the ratio of Firefly/Renilla luciferase activities normalised to empty miReport vector (miReport) ± S.E.M. (n = 9, * = *P*<0.001 by Student *t*-test).

In order to establish whether ECE-1 expression is subject to post-transcriptional regulation via its 3′UTR in cancer cells, the entire 3′UTR (2710 bp) was cloned into a luciferase reporter vector. A variety of cell lines were subsequently transfected with the ECE-1 UTR-luciferase construct or the empty luciferase vector (Figure 1). In all cell lines tested, the full-length 3′UTR caused a significant reduction in luciferase activity compared to empty vector as measured by luciferase reporter assay. The greatest effect was seen in PC-3 prostate cancer cells (86% reduction in luciferase activity) but transfection of RWPE-1, CHO and Huh7 cells also showed a significant reduction, suggesting this is not a cancer- or cell-line specific phenomenon.

### The ECE-1 3′UTR Reduces ECE-1 Protein Expression

**Figure 2:**
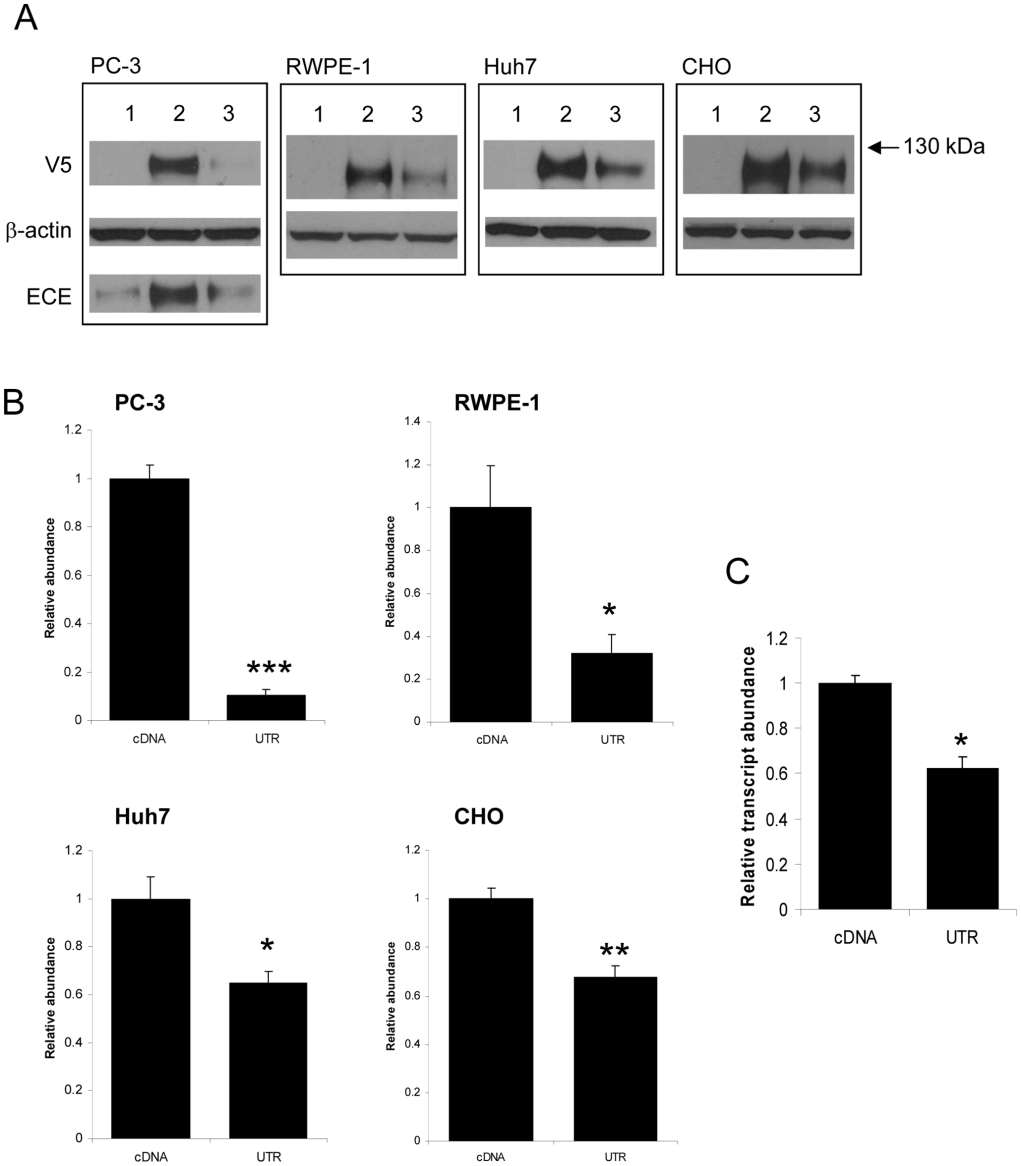
The 3′-UTR of ECE-1 represses ECE-1 protein expression. PC-3, RWPE-1, Huh7 and CHO cells were transiently transfected with empty vector (1), V5-tagged ECE-1c cDNA (2) or V5-tagged ECE-1c cDNA with full-length 3′ UTR (3) and immunoblotted for V5 (A). β-actin was used as a loading control. The PC-3 blot was reprobed for ECE-1 expression. Data represent the ratio of V5:actin expression ± S.E.M. (B; n = 3, * = *P*<0.05, ** = *P*<0.01, *** = *P*<0.001 by Student *t*-test). Real-time PCR analysis of transcript abundance in transfected PC-3 cells shown in A. V5 expression was normalised to neomycin expression (transfection control) and U6 expression (endogenous control). n = 3, * = *P*<0.005 by Student *t*-test (C).

We next examined the effect of the ECE-1 3′UTR on ECE-1 protein expression. ECE-1c cDNA with an N-terminal V5 tag was cloned into the pcDNA3 mammalian expression vector with or without the full-length 3′UTR. Use of the V5 tag allowed detection only of heterologously expressed ECE-1; the majority of cell lines endogenously express ECE-1. ECE-1c cDNA was used because it is the most widely expressed isoform, and is generally expressed at the highest level of all the isoforms [6]. A variety of cell lines were transfected with V5-cDNA (without UTR) or V5-UTR (with UTR) and immunoblotted for V5 (Figure 2A). In all cases, the full-length UTR significantly reduced ECE-1 protein expression. Again, the greatest decrease was seen in PC-3 cells (Figure 2B).

Real-time PCR was then used to examine V5-tagged transcript level (using primers which do not detect endogenous ECE-1 expression) in the transfected PC-3 cells, and showed that transcript level is significantly downregulated by the full-length UTR (Figure 2C).

### The ECE-1 3′UTR Contains Alternative Polyadenylation Sites

It has recently been reported that cancer cell lines often express high levels of transcripts with truncated 3′ UTRs, which usually result from alternative cleavage and polyadenylation [15]. Cleavage and polyadenylation is required for maturation of most mRNA transcripts. Approximately 54% of human genes contain alternative polyadenylation sites [17]; AAUAAA is the canonical polyadenylation signal, but variants are also used. These variants, including AAGAAA, AAUACA, AAUGAA and UUUAAA, are generally used as proximal polyadenylation signals rather than the polyadenylation signal for the longest isoform [17]. Bioinformatic analysis revealed the 3′ UTR of ECE-1 contains 6 such APA sites of varying predicted strength (Table 1).

**Table 1:** The ECE-1 3′UTR contains 6 putative APA sites.

Sequence	Position in 3′UTR	Score
TTTAAA	352 bp	0.671
AATACA	366 bp	0.732
TTTAAA	397 bp	0.771
ACTAAA	1182 bp	0.113
AAGAAA	1357 bp	0.157
AATGAA	2538 bp	0.814
AATAAA	2686 bp	0.157

*In silico* interrogation (http://dnafsminer.bic.nus.edu.sg/) of the ECE-1 3′UTR sequence revealed the presence of six potential proximal polyadenylation signals.

### Cancer Cells Express ECE-1 mRNA Transcripts with Truncated 3′ UTRs

**Figure 3:**
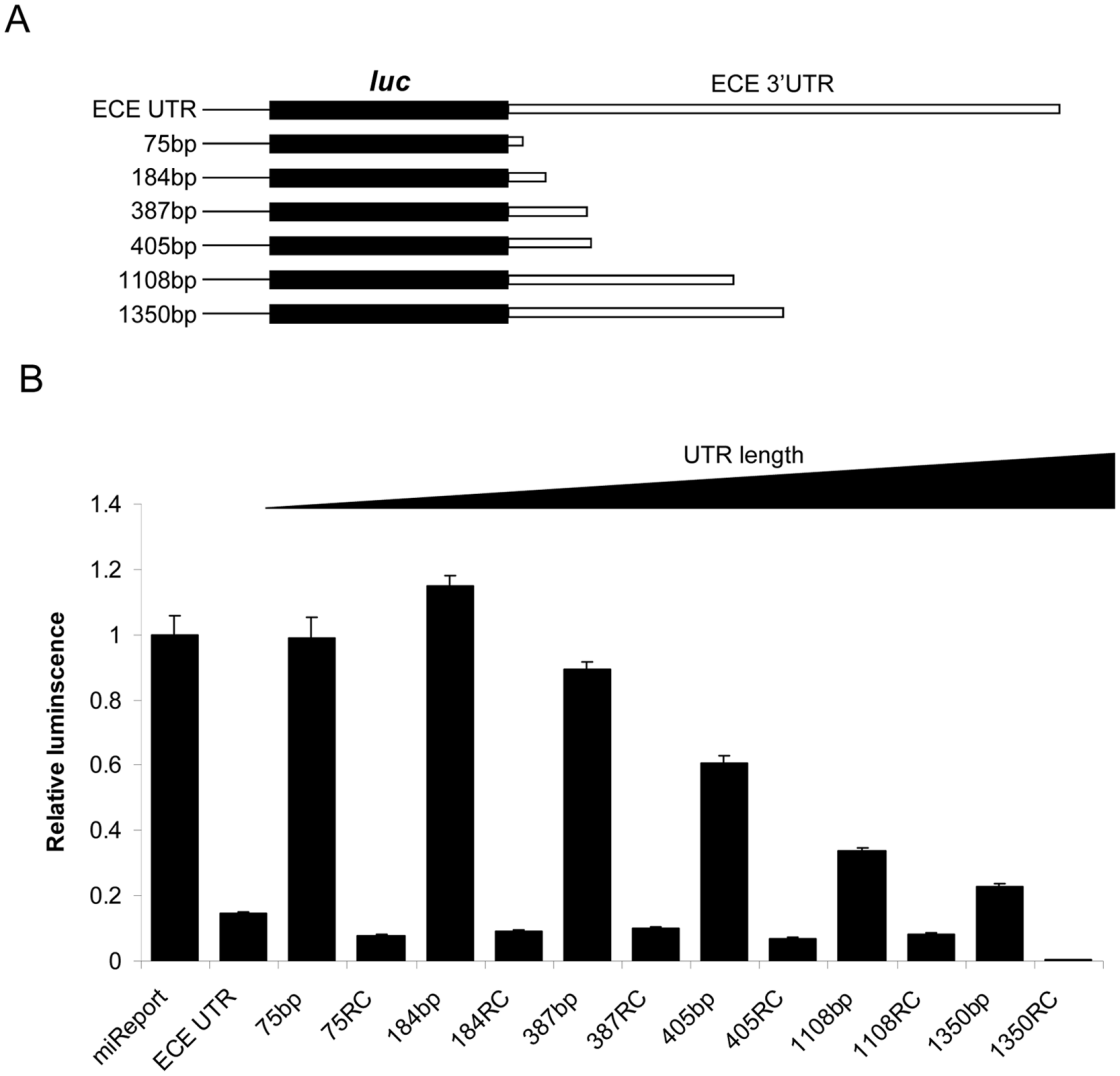
The effect of the ECE-1 3′UTR on luciferase activity is due to length not specific sequence. Schematic representation of the truncated UTRs identified by 3′RACE which were then cloned downstream of the Firefly luciferase gene in pMIR-REPORT (A). Empty pMIR-REPORT, full-length ECE UTR, the six truncated UTRs and the equivalent six truncated UTRs controlled for length (RC) were transfected into PC-3 cells. Activities of Firefly and Renilla luciferase (endogenous control) were assayed 48 h post transfection. Data represents the ratio of Firefly/Renilla activities normalised to empty pMIR-REPORT vector (miReport) ± S.E.M. (n = 9) (B).

To investigate if the predicted APA sites are functional and produce truncated UTRs, 3′RACE (rapid amplification of cDNA ends) was performed on RNA isolated from PC-3 cancer cells. Six truncated transcripts, as well as the full-length UTR, were isolated (represented in Figure 3A). These truncations matched closely, but not completely, to the putative APA sites identified by bioinformatics analysis.

The truncated UTRs identified by 3′RACE were cloned into a luciferase reporter vector and transfected into PC-3 cells to assess the effect of these physiologically relevant truncations on protein translation. To control for non-specific functions of UTR length the reverse complement (RC) of the UTR segment present in the full-length but not truncated UTR was cloned immediately downstream of the truncated UTR, so that all RC constructs contained 2710 nucleotides.

The resulting constructs were then transfected into PC-3 cells and analysed by luciferase reporter assay (Figure 3B). The effect of the UTR on luciferase activity appears to be solely due to length, since the same specific sequence is present in both the truncated construct and the RC construct. The UTR of 184 bp significantly increased luciferase activity by 14.7%, but UTRs of 387 bp or longer decreased luciferase activity.

### 3′UTR Length Affects ECE-1 Protein Expression

**Figure 4:**
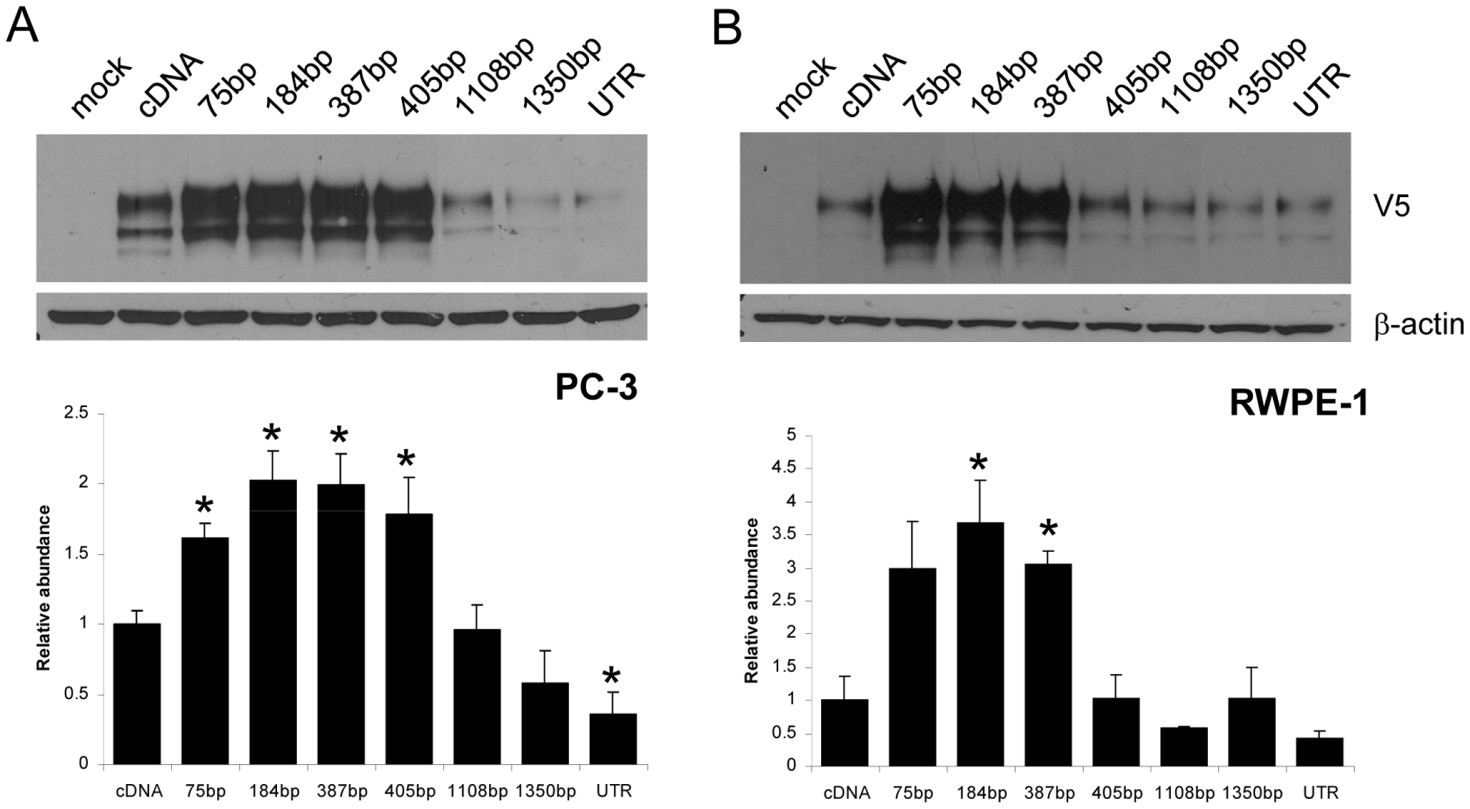
The effect of the ECE-1 3′UTR on ECE protein expression depends on UTR length. PC-3 (A) or RWPE-1 (B) cells were transfected with V5-tagged ECE-1c cDNA only or with the truncated or full-length UTR cloned immediately downstream for 48 h. Total lysates (30 mg protein) were immunoblotted for V5; b-actin was used as a loading control. Data represent the ratio of V5:actin expression ± S.E.M. n = 3, * = *P*<0.05 by Student *t*-test.

**Figure 5:**
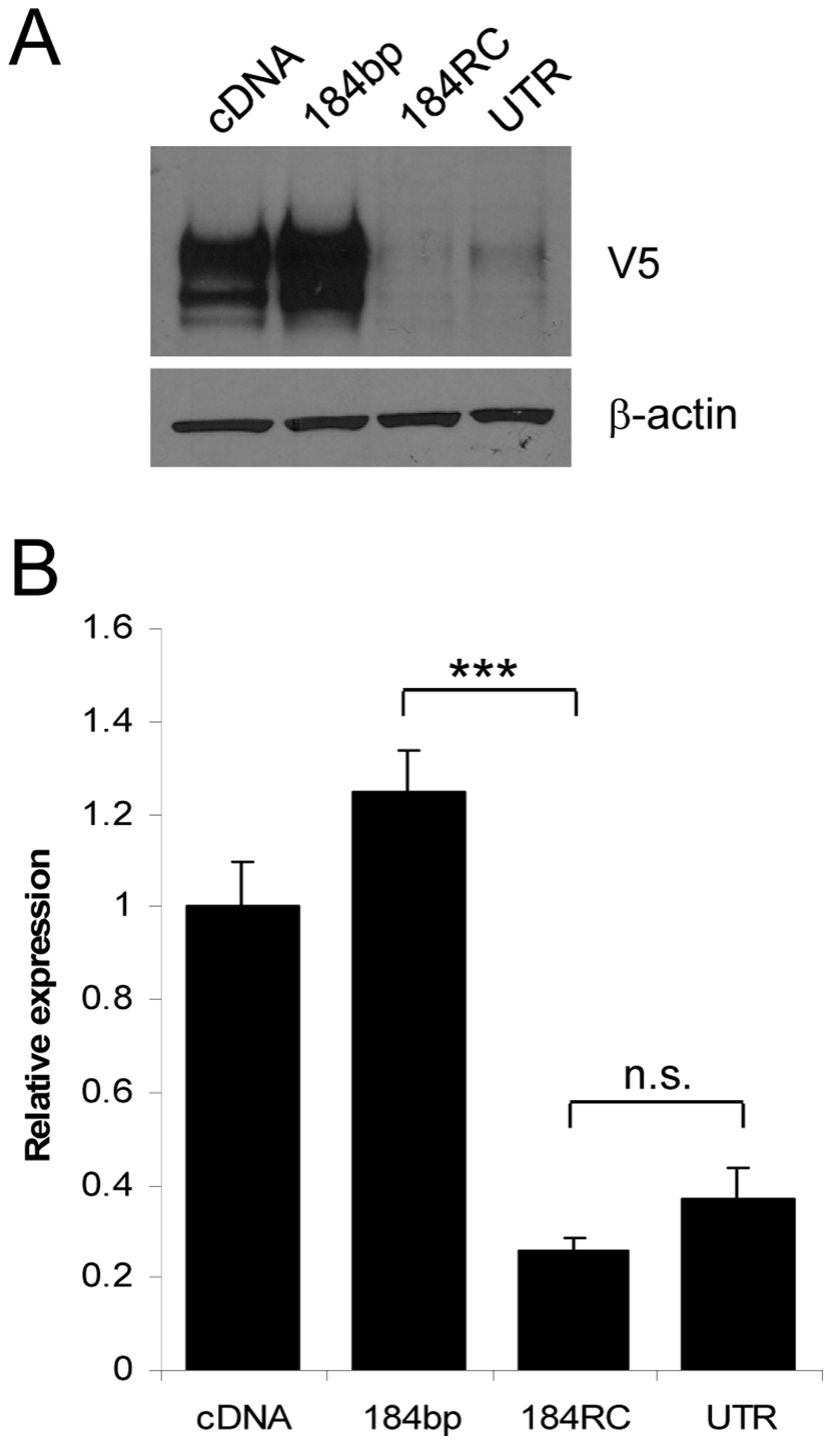
The effect of the UTR on ECE protein expression is due to length not specific sequence. PC-3 cells were transfected with V5-tagged cDNA, V5 cDNA plus 184 bp UTR, V5 cDNA plus 184 bp+RC, or V5 cDNA plus full-length UTR for 48 h. Total lysates (30 mg) were immunoblotted for V5; b-actin was used as a loading control (A). Data represent the ratio of V5:actin expression normalised to cDNA ± S.E.M. n = 3, *** = P<0.001 by Student t-test, n.s = not significant (B).

Having established that 3′UTR length strongly influences translatability in a luciferase reporter system, we next analysed the effect of the truncated UTRs on ECE-1 protein expression. Each truncated 3′UTR identified from 3′RACE analysis was cloned immediately downstream of the V5-tagged cDNA, and the resulting constructs transfected into PC-3 or RWPE-1 cells (Figure 4A and B). As observed using the reporter assay, an increase in UTR length up to 184 bp increased ECE-1 expression, in keeping with the findings of the luciferase reporter assay. UTRs of 387 bp or 405 bp both increased protein expression beyond that of cDNA only, but to a lesser extent. 3′UTR lengths of 1108 bp, or longer, decreased protein expression significantly below that of cDNA alone. Replacement of the deleted region of the 3′UTR with its reverse complement restored ECE-1 protein expression to levels similar to that of the wild-type, suggesting this observation is not due to specific sequence motifs within the 3′UTR (Figure 5A, B).

### Removal of the APA Sites Decreases Luciferase Activity

**Figure 6:**
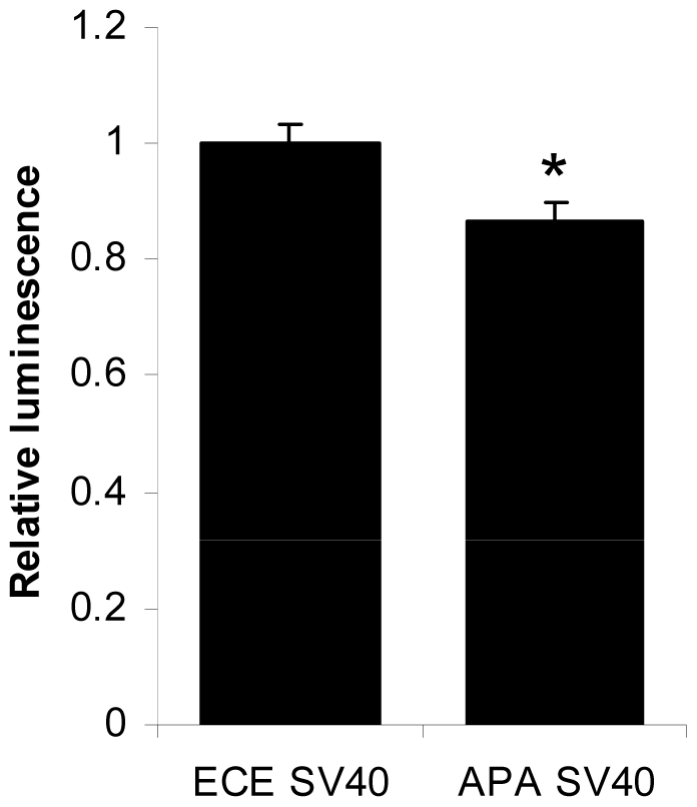
Removal of the APA sites reduces luciferase activity. The SV40poly(A) tail sequence was deleted from the wild-type ECE-UTR miReport construct (ECE SV40). The six APA sites were then all abolished by site-directed mutagenesis (APA SV40). Constructs were transfected into PC-3 cells for 48 h. Data represent the ratio of Firefly/Renilla luciferase activities normalised to ECE SV40± S.E.M. (n = 9, * = *P*<0.05 by Student *t*-test).

As discussed above, the ECE-1 UTR contains six APA sites in addition to the distal polyadenylation signal. To assess the effect of these APA sites the SV40 poly(A) signal was deleted from the vector to prevent the strong poly(A) signal overriding the endogenous polyadenylation signals. To assess the effect of the APA sites, all six were then mutated. The wild-type UTR construct and the mutated construct were then transfected into PC-3 cells and luciferase activity analysed (Figure 6). Removal of the APA sites caused a small but significant decrease of 13.5% in luciferase activity.

## Discussion

It has been previously reported that ECE-1 is upregulated in a variety of cancers, but the mechanism of this upregulation has not been elucidated. We demonstrate here, for the first time, that the 3′UTR plays a significant role in regulating the expression of ECE-1.

A number of recent reports have identified widespread alterations in 3′UTR length in proliferating and malignant cells, generally manifested in the form of transcript truncations [17], [18]. This truncation may free transcripts from regulation by factors, protein or RNA (particularly microRNA), which interact with the 3′UTR and influence protein expression, or influence their translocation from the nucleus. In a subset of mantle cell lymphoma, for example, a truncated Cyclin D1 transcript is over-represented, leading to an increase in Cyclin D1 protein expression [19]. Although the mechanisms underlying transcript shortening in distinct physiological and pathophysiological states is unclear, a variety of mechanisms are likely to be involved, including genomic deletions, point mutations and the utilisation of alternative polyadenylation (APA) signals. Polyadenylation is a key step in the maturation of transcripts; most genes contain strong polyadenylation signals which direct transcript cleavage and the addition of a poly(A) tail. A number of genes, however, also contain APA signals which, in certain contexts, may be utilised to generate a truncated transcript. In cancer cells, for example, it is thought that levels of 3′ processing factors are frequently altered (16) and these global changes may lead to changes in poly(A) site selection. Cytoprotective heat shock protein 70 (Hsp70), for example, is post-transcriptionally regulated via alternative polyadenylation (APA) and miRNAs. APA, induced by heat shock stimulus, causes accumulation of transcripts with truncated 3′UTRs. This shortening of the 3′UTR both relieves the translational repression observed with the long 3′UTR, and removes binding sites for suppressive miRNAs, causing increased expression [20]. It has been shown by systematic analysis of human and mouse transcriptomes that short 3′UTR isoforms are relatively more abundant when genes are highly expressed and that poly(A) site choice is coupled to transcriptional activity [21].

Here we show that truncations in the ECE-1 3′UTR lead to increased ECE-1 mRNA and protein expression. In the course of this study it was reported that nitric oxide (NO) causes a decrease in ECE-1 expression, via destabilization of the transcript via the 3′UTR. NO acts through a cGMP-dependent mechanism and a region of the 3′UTR containing regulatory elements involved in this mechanism was identified [10]. A study of hepatic wound healing detected upregulation of ECE protein expression and identified two 3′UTR-binding proteins which cause stabilization of the transcript [11]. Although in these studies the rat ECE-1 3′UTR was used, which differs substantially in both length and sequence to the human 3′UTR, this does suggest a conserved role for the 3′UTR in regulating the expression of ECE-1.

Here we provide evidence that the ECE-1 3′UTR contains APA sites, and that these may be used to generate truncated transcripts in prostate cancer cells. Heterologous expression of constructs containing truncations corresponding to those identified in PC cells led to enhanced protein expression, and mutation of the APA sequences led to a decrease in expression in a reporter assay, most likely due to an increase in expression of the full length 3′UTR resulting from the removal of APA-mediated transcript cleavage.

The mechanisms underlying the observed increase in ECE-1 expression on truncation of its 3′UTR remain to be determined. The data presented here suggest that this phenomenon is purely a function of transcript length rather than the result of elimination of specific sequences; replacement of the deleted regions with the reverse complement restored the repression of gene expression. This is at odds with the recent reports in rats, in which specific regulatory regions in the 3′UTR were identified [10], [11], and suggests that different mechanisms may exist in different cell types and/or species. Work is ongoing to identify the precise molecular mechanisms underlying the 3′UTR-induced suppression of ECE-1 expression, but our findings suggest this may be a novel mechanism responsible for ECE-1 over-expression in cancer cells and may therefore represent a novel therapeutic target.
